# Biocompatibility of Direct 3D-Printed Clear Aligner Resins: A Comparative In Vitro Cytotoxicity Analysis

**DOI:** 10.4317/jced.64159

**Published:** 2026-05-29

**Authors:** Nida Tutka, Betül Gülhan Çakir, Ahmet Murat Artuç

**Affiliations:** 1 Istanbul Aydın University, Faculty of Dentistry, Department of Orthodontics, Istanbul, Türkiye

## Abstract

**Background:**

This study comparatively evaluated the mechanical properties of four commercially available, directly three-dimensional (3D) printed clear aligner materials by analyzing tensile behavior, flexural properties, and surface hardness.

**Materials and Methods:**

Four direct-print clear aligner resins were included: CRS, LuxCreo, Rayform 4D clear aliner resin, and PowerResins clear smile resin. Specimens were fabricated according to the manufacturers' protocols with a standardized thickness of 0.70 mm and 50 m layer thickness in vertical orientation. Tensile (n = 5), three-point bending (n = 5), and Shore D hardness (n = 10) tests were performed at room temperature (23°C) at the accredited laboratories of TÜBTAK Marmara Research Center. Statistical analysis was performed using one-way analysis of variance followed by Sidak's multiple comparisons test (p &lt; 0.05).

**Results:**

Significant differences were found among all materials for all tested parameters (p &lt; 0.0001). PowerResins showed the highest elastic modulus (2187.7 ± 315.8 megapascals [MPa]), tensile strength (51.5 ± 2.9 MPa), flexural modulus (1609.7 ± 95.6 MPa), flexural strength (71.9 ± 2.9 MPa), and Shore D hardness (87.3 ± 0.9), indicating superior rigidity. LuxCreo demonstrated the highest elongation at break (118.4 ± 10.7%), suggesting greater flexibility and ductility. Rayform 4D showed intermediate mechanical behavior, whereas CRS generally exhibited lower flexural resistance.

**Conclusions:**

Directly printed clear aligner materials exhibit significant variability in mechanical properties. PowerResins may be more suitable for cases requiring greater rigidity and force delivery, whereas LuxCreo may be preferable when flexibility and patient comfort are prioritized. Material selection should be based on specific orthodontic treatment objectives and clinical biomechanical requirements.

## Introduction

Clear aligner therapy has become an integral component of contemporary orthodontic practice due to its aesthetic advantages, enhanced patient comfort, and compatibility with digital workflows (1). Conventionally, aligners are manufactured using thermoforming techniques, in which a thermoplastic sheet is adapted over a three-dimensional (3D)-printed dental model. Although this method is clinically well established, it presents several limitations, including potential material deformation during thermoforming, variability in thickness, and multiple processing steps that may influence the final properties of the appliance (2). Recent advances in additive manufacturing have enabled the direct fabrication of clear aligners using 3D printing technologies. This emerging approach eliminates the need for intermediate models and thermoforming procedures, allowing for improved control over aligner geometry and potentially enhancing manufacturing efficiency (3). However, directly 3D-printed aligners are composed of photopolymerizable resin systems, and their final properties are strongly influenced by material composition, printing parameters, and post-processing protocols. These factors may significantly affect the degree of polymerization and the release of residual monomers or degradation products (4 , 5). Biocompatibility is a critical consideration for aligner materials, as these appliances remain in prolonged contact with teeth, gingival tissues, and oral mucosa, typically for more than 20 hours per day. The oral environment is dynamic, characterized by fluctuations in temperature, pH, humidity, and enzymatic activity, all of which may contribute to material degradation and the release of biologically active substances (6). These released compounds may interfere with cellular metabolism, membrane integrity, and overall cell viability, making cytotoxicity assessment an essential component in the evaluation of dental biomaterials (7 , 8). Previous studies investigating the biological effects of aligner materials have reported heterogeneous findings. While some studies have demonstrated minimal or no cytotoxic and estrogenic effects under controlled conditions, others have reported reductions in cell viability and alterations in cellular behavior depending on the material type, extraction conditions, and testing protocols (9 , 10). The biological response appears to be influenced by multiple factors, including extract concentration, exposure duration, and environmental conditions, with saliva potentially exerting a protective effect (11). Furthermore, recent evidence suggests that post-processing procedures, particularly post-curing, play a crucial role in determining the biocompatibility of 3D-printed resins, as insufficient polymerization may increase the release of unreacted components and consequently enhance cytotoxicity (12 , 13). Despite the increasing clinical adoption of directly printed aligners, the current literature regarding their cytotoxicity profiles remains limited and, in some cases, inconsistent. Additionally, variations in resin composition and manufacturer-recommended processing protocols may lead to significant differences in biological performance among materials. Therefore, comparative studies evaluating multiple direct 3D-printed aligner resins under standardized conditions are essential to better understand their biological safety (14 , 15). In vitro cytotoxicity testing, particularly through indirect extract methods in accordance with ISO 10993 standards, provides a reliable approach for assessing the biological effects of substances released from polymeric materials (16). Among these methods, the MTT assay is widely used to evaluate cellular metabolic activity and viability, offering a quantitative measure of material-induced cytotoxicity (17). Accordingly, the aim of the present study was to evaluate and compare the cytotoxic effects of different commercially available direct 3D-printed clear aligner resins using an indirect extract method on L929 fibroblast cells. The null hypothesis was that no significant difference in cytotoxicity would be observed among the tested materials.

## Materials and Methods

1. Study design This in vitro experimental study was designed to evaluate the cytotoxic effects of different direct three-dimensional (3D)-printed clear aligner resins. Cytotoxicity was assessed using an indirect extract method in combination with the MTT assay, in accordance with ISO 10993-5 and ISO 10993-12 standards (17). 2. Materials Five commercially available direct 3D-printed clear aligner resins were included in this study: LuxCreo - LuxCero (LuxCreo Inc., California, USA) CRS - Custom Aligner Resin (Custom Resin Solutions, Turkey) enertek - Clear-A (enertek, Turkey) PowerResins - Clear Smile Resin (3BFAB, Turkey) Rayform - 4D Clear Aligner Resin (Rayform Co., Ltd., South Korea) All materials were processed according to the manufacturers' recommended protocols, and batch numbers were recorded. All specimens were fabricated and processed under strictly standardized conditions, including identical design parameters, printing settings, and post-processing protocols, to ensure consistency and comparability among groups. Samples were designed using CAD software (Tinkercad, Autodesk Inc., USA). For each material, five square specimens measuring 20 × 20 mm with a thickness of 0.7 mm were fabricated. All samples were produced under standardized printing conditions, including a layer thickness of 50 µm, vertical orientation, and without support structures. From each square specimen, five circular discs with a diameter of 4 mm were obtained. Accordingly, a total of 25 discs were prepared for each material, resulting in five extract samples per group (each consisting of five discs) and an overall total of 125 discs. 3. Post-Processing Procedure All materials were subjected to post-processing according to manufacturer instructions: LuxCero: Provided fully processed and polymerized within a closed system workflow. PowerResins (Clear Smile Resin): Final curing performed using Otoflash G-171 under nitrogen (N2) atmosphere (1000×2 flashes). CRS (Custom Aligner Resin): Cleaned using 98% isopropyl alcohol (IPA) with multi-step washing and ultrasonic cleaning, followed by air drying. Final curing was performed using a Dentafarm Photopol UV unit (120 W, 2 + 2 minutes, inverted), under nitrogen atmosphere. enertek (Clear-A): Cleaned via centrifugation, followed by UV curing in glycerin medium and subsequent ultrasonic cleaning at 80-90°C. Rayform (4D Clear Aligner Resin): Cleaned with IPA, air-dried, and UV-cured (Phrozen unit) for 30 minutes. 4. Extract Preparation and cell culture Cytotoxicity was evaluated using an indirect extract method. For each extract, five discs were immersed in DMEM culture medium and incubated at 37°C for 72 hours. Following incubation, the extracts were sterilized by filtration and subsequently applied to the cell cultures. L929 mouse fibroblast cells were used for cytotoxicity assessment. The cells were cultured in DMEM medium supplemented with 10% fetal bovine serum and 1% antibiotic, and maintained at 37°C in a humidified atmosphere containing 5% CO2. 5. MTT Cytotoxicity Assay Cells were seeded into 96-well plates and allowed to reach appropriate confluency. The culture medium was then removed, and the prepared material extracts were applied to the cells. Following 72 hours of incubation, MTT reagent was added to each well, and the formation of formazan crystals was allowed to occur. Subsequently, the crystals were dissolved, and absorbance values were measured at 570 nm using a spectrophotometer. Cell viability was expressed as a percentage relative to the negative control. For control conditions, the negative control group consisted of DMEM / DMEM-F12 culture medium, while the positive control group was treated with DMSO. 6. Statistical analysis Statistical analyses were performed using GraphPad Prism software (version 9.0, GraphPad Software, USA). Continuous variables were expressed as mean ± standard deviation (SD). Normality of data distribution was assessed using the Shapiro-Wilk test. Differences among groups were analyzed using one-way analysis of variance (one-way ANOVA). When a significant difference was detected, pairwise comparisons between groups were performed using Sidak's multiple comparisons test. A p-value of &lt;0.05 was considered statistically significant. Cell viability data were normalized to the mean value of the negative control group and expressed as percentage (%). Prior to the study, sample size estimation was performed using G*Power software (version 3.1.9.7). Based on a one-way ANOVA model comparing five independent groups, the significance level was set at = 0.05 and the statistical power (1) at 0.80. Considering the expected pronounced differences among materials in in vitro cytotoxicity studies, a large effect size (f = 0.90) was assumed. Under these assumptions, the minimum total sample size was calculated as 25. In the present study, four replicates per group were used, resulting in a total of 20 measurements. The corresponding achieved power for this sample size was calculated as 0.91.

## Results

**Figure 1:**
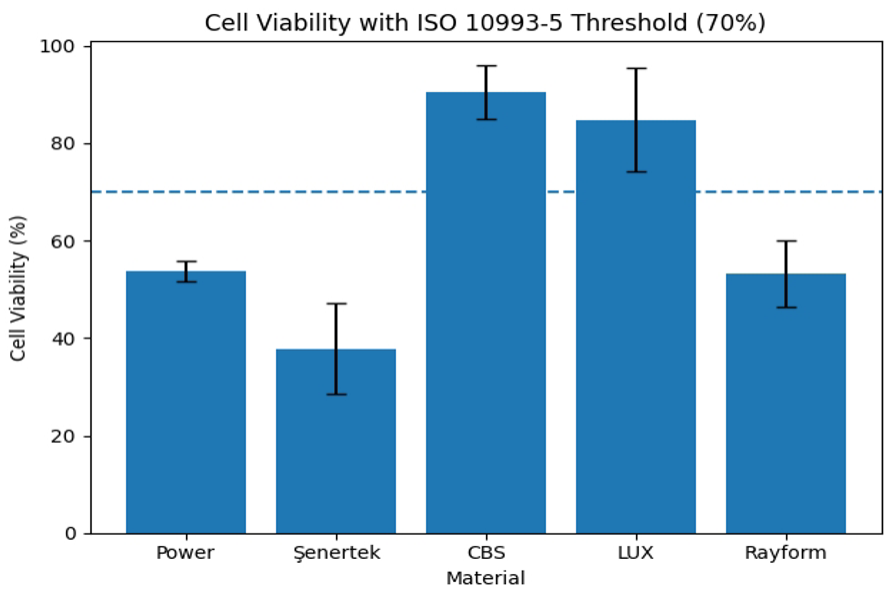
Cell viability (%) of L929 fibroblast cells exposed to different direct 3D-printed aligner materials. Data are presented as mean ± standard deviation (n = 5). The dashed horizontal line represents the ISO 10993-5 cytotoxicity threshold (70% cell viability). Materials above this threshold are considered non-cytotoxic (CBS and LUX), whereas those below are classified as cytotoxic (Power, Şenertek, and Rayform).

Cell viability of L929 fibroblast cells exposed to different direct 3D-printed materials was evaluated using absorbance measurements and expressed as a percentage relative to the negative control. One-way ANOVA revealed a statistically significant difference among the groups (F(6,21) = 98.40, p &lt; 0.0001, R² = 0.9657), indicating a strong effect of material type on cell viability. Post hoc analysis using Sidak's multiple comparisons test demonstrated that the enertek group had significantly lower cell viability compared to all other material groups (p &lt; 0.05 for all comparisons). In contrast, CBS and LUX groups exhibited significantly higher cell viability than the Power, enertek, and Rayform groups (p &lt; 0.0001). No statistically significant difference was observed between the Power and Rayform groups (p &gt; 0.9999) or between the CBS and LUX groups (p = 0.9322). The mean cell viability values (mean ± SD, n = 5) were as follows: Power (53.61 ± 2.10%), enertek (37.84 ± 9.20%), CBS (90.47 ± 5.60%), LUX (84.78 ± 10.60%), and Rayform (53.28 ± 6.80%). The negative control group showed 100.00 ± 2.10% cell viability, whereas the positive control group demonstrated markedly reduced viability (7.95 ± 0.70%). According to ISO 10993-5 criteria (70% cell viability), CBS and LUX materials were classified as non-cytotoxic, whereas Power, enertek, and Rayform materials were classified as cytotoxic, (Fig. 1).

1

All data were normalized to the negative control and are presented as mean ± standard deviation, (Table 1).

Table 1

## Discussion

The present study evaluated the cytotoxic effects of different direct 3D-printed clear aligner resins using a standardized indirect extract method. The findings revealed a clear material-dependent variation in cytotoxicity, with CBS and LUX classified as non-cytotoxic, whereas Power, enertek, and Rayform exhibited cytotoxic effects according to ISO 10993-5 criteria. Among all tested materials, the enertek resin demonstrated the lowest cell viability, indicating a pronounced biological response. The observed differences in cytotoxicity can be attributed to several factors, most notably resin composition and post-processing protocols (18). Although all materials were processed according to manufacturer recommendations, the post-processing workflows varied considerably among groups. Variations in cleaning methods (centrifugation versus isopropyl alcohol washing), curing environments (nitrogen atmosphere versus conventional UV), and curing durations may have influenced the degree of polymerization and, consequently, the release of residual monomers. Previous studies have shown that insufficient or suboptimal post-curing significantly increases the release of unreacted components, which is directly associated with higher cytotoxicity levels (19). This is consistent with the markedly reduced cell viability observed in the enertek group in the present study. Another important consideration is the standardized sample design and extraction protocol used in this study. All materials were fabricated with identical dimensions and printed under consistent parameters, thereby minimizing variability related to manufacturing conditions. In addition, the use of a controlled extract preparation protocol enabled a reproducible assessment of substances released from the materials. Therefore, the differences observed in cytotoxicity are more likely attributable to intrinsic material properties rather than experimental variability. The results of the present study are consistent with previous reports indicating that 3D-printed aligner materials exhibit heterogeneous biocompatibility profiles. Several studies have demonstrated that certain resin systems may reduce cell viability depending on extraction concentration and exposure conditions, whereas others remain within non-cytotoxic thresholds (20). This variability underscores the importance of evaluating each material individually rather than generalizing the biological safety of all 3D-printed aligner systems. In addition to material composition, the interaction between extraction conditions and biological response should also be considered. It has been reported that eluates prepared under different environmental conditions may produce varying biological outcomes, with saliva potentially exerting a protective effect against cytotoxic components (21). Although a standardized extraction medium was used in the present study, intraoral factors such as salivary buffering capacity and enzymatic activity may influence the biological impact of released substances. The findings also highlight the potential role of dose-dependent effects of leached components. Even under identical extraction conditions, certain materials may release higher concentrations of residual monomers or degradation products, leading to increased cytotoxicity. This may explain why the Power and Rayform groups exhibited moderate cytotoxicity, whereas CBS and LUX maintained high levels of cell viability. Similar dose-dependent cytotoxic responses have been reported in previous studies evaluating both 3D-printed and thermoformed aligner materials (20). Interestingly, the literature also includes studies reporting no cytotoxic or estrogenic effects for certain 3D-printed aligner materials under controlled conditions (22). These discrepancies may be attributed to differences in resin formulations, extraction protocols, aging conditions, and analytical methods. Therefore, the variability observed in the present study is consistent with the heterogeneous nature of the existing literature. Another critical factor is the potential impact of intraoral aging and long-term exposure. Previous studies have shown that simulated oral aging can alter the chemical structure and surface properties of 3D-printed resins, leading to increased release of degradation products over time (19). In the present study, cytotoxicity was evaluated under short-term extraction conditions; however, the cumulative effects of repeated aligner use in clinical settings should be considered when interpreting these findings. From a clinical perspective, the identification of materials such as CBS and LUX with high cell viability suggests that certain direct 3D-printed resins may offer a more favorable biological profile for orthodontic applications. In contrast, materials exhibiting lower cell viability, particularly enertek, may require further optimization of processing protocols or additional biocompatibility evaluation prior to widespread clinical use. Despite the strengths of the present study, including standardized manufacturing parameters and controlled extraction conditions, several limitations should be acknowledged. First, the study was conducted under in vitro conditions, which cannot fully replicate the complex oral environment. Additionally, the use of a single cell line (L929 fibroblasts) and a single assay (MTT) may limit the generalizability of the findings to broader biological responses. As the MTT assay primarily reflects mitochondrial activity, it may not fully capture other biological responses, such as inflammatory signaling, oxidative stress, or membrane integrity. Therefore, future studies incorporating multiple cell types and complementary analytical methods would provide a more comprehensive evaluation of material biocompatibility.

## Conclusions

The present study demonstrated that the cytotoxicity of direct 3D-printed clear aligner resins varies significantly depending on the material. CBS and LUX resins exhibited favorable biocompatibility profiles and were classified as non-cytotoxic according to ISO 10993-5 criteria, whereas Power, enertek, and Rayform materials showed cytotoxic effects under the tested conditions. These findings indicate that direct 3D-printed aligner materials cannot be considered biologically equivalent, and their cytotoxic behavior may be influenced by factors such as material composition and post-processing protocols. From a clinical perspective, the selection of aligner materials with proven biocompatibility is essential to ensure patient safety during long-term intraoral use. Further research is needed to evaluate the long-term biological effects of these materials under conditions that more closely simulate the oral environment. In addition, the relatively limited number of replicates used in this study may reduce the statistical robustness of the findings. Furthermore, the absence of long-term aging or degradation simulations represents an additional limitation, as intraoral conditions may alter material properties and cytotoxic behavior over time. Therefore, future studies incorporating long-term exposure models and simulated oral aging conditions are warranted to better reflect clinical scenarios.

## Figures and Tables

**Table 1:** Cell viability (%) values of direct print materials.

Group	Measurement 1 (%)	Measurement 2 (%)	Measurement 3 (%)	Measurement 4 (%)	Mean ± SD (%)
Power	54.63	54.17	55.11	50.54	53.61 ± 2.10
Şenertek	29.23	29.79	46.23	46.11	37.84 ± 9.20
CBS	83.77	95.56	87.56	94.99	90.47 ± 5.60
LUX	86.72	99.48	78.29	74.63	84.78 ± 10.60
Rayform	55.45	60.66	52.85	44.15	53.28 ± 6.80
Control (-)	97.81	98.53	101.71	101.91	100.00 ± 2.10
Control (+)	8.63	7.90	8.31	6.94	7.95 ± 0.70

1

## Data Availability

The data supporting the findings of this study are available from the corresponding author upon reasonable request.
